# Enhanced Recovery After Surgery (ERAS) Versus Standard Postoperative Care in Elective Craniotomy for Brain Tumor Excision: A Comprehensive Review of Protocol Differences, Clinical Outcomes, and Implementation Challenges

**DOI:** 10.7759/cureus.113797

**Published:** 2026-08-01

**Authors:** Mateo Serrano Pinzón, Sadaf Afshar, Jocelyn N Wensel, Amritha V Manikantan, Nazariy Matiytsiv, Alkama Hita, Amel Zilani, Arnab Saha, Anderlie Pierre, Adrian Joseph, Anyagwa Onyekachi

**Affiliations:** 1 Neurological Surgery, Pontificia Universidad Javeriana, Bogotá, COL; 2 Neurology, Medical University of Lublin, Lublin, POL; 3 Medical Education, International University of Health Sciences (IUHS), Basseterre, KNA; 4 Neurological Surgery, St. George's University, St. George's, GRD; 5 Neurological Surgery, Ivano-Frankivsk National Medical University, Ivano-Frankivsk, UKR; 6 Neurological Surgery, Bogomolets National Medical University, Kyiv, UKR; 7 Neurosurgery, Shadan Institute of Medical Sciences, Hyderabad, IND; 8 Neurosurgery, King's College Hospital NHS Foundation Trust, Kent, GBR; 9 Neurological Surgery, Université Notre Dame d'Haïti, Port-au-Prince, HTI; 10 Medicine and Surgery, Royal College of Surgeons in Ireland, Dublin, IRL; 11 School of Medicine, New Vision University, Tbilisi, GEO

**Keywords:** alternative anesthesia, brain tumor, elective craniotomy, enhanced recovery after surgery (eras), length of stay, standard postoperative care

## Abstract

Standard postoperative care following elective craniotomy traditionally includes close monitoring in an ICU setting, use of opioid-based analgesia, delayed initiation of oral intake, and prolonged bed rest. These practices may contribute to a longer recovery period, a higher risk of complications such as infections or deep vein thrombosis (DVT), and lower patient satisfaction.

On the other hand, Enhanced Recovery After Surgery (ERAS) protocols offer a more structured and proactive approach. It incorporates preoperative counseling, carbohydrate loading (if appropriate), avoidance of prolonged fasting, and standardized anesthetic techniques that prefer shorter-acting agents. Postoperatively, ERAS prioritizes early removal of catheters and drains, early enteral diet, and rapid mobilization - all designed to help patients recover more efficiently. Across the literature reviewed here, ERAS is associated with concrete gains over standard care: postoperative nausea and vomiting falling from roughly 28.0% to 9.2% in one cohort, a Karnofsky Performance Status improvement of approximately 10 points sustained at 30 days (pooled mean difference 6.68 points), patient satisfaction of 92.2% versus 86.8%, ICU length of stay reduced to under one day, and per-patient cost reductions of $1,044-$4,026. Hospitals must undertake protocol development, arrange training, and ensure flexibility to adapt ERAS elements to each patient's clinical needs; implementation remains uneven across economic and resource settings, particularly in low- and middle-income countries. As interest in ERAS grows, further research and education will be essential to support wider and more effective practice across neurosurgical practices.

## Introduction and background

Elective craniotomy is a common neurosurgical procedure for conditions such as brain tumors, vascular malformations, epilepsy, and other intracranial abnormalities. Postoperative recovery is often prolonged, impacted by pain, nausea, neurological deficits, and delayed mobilization, which can contribute to longer hospital stays and higher complication rates [1-2]. Standard postoperative care is variable and largely institution-dependent, sometimes lacking a structured, multidisciplinary framework, which may affect the quality and duration of recovery.

In 1997, Kehlet introduced the multimodal (*fast-track*) rehabilitation concept that underlies Enhanced Recovery After Surgery (ERAS), aiming to reduce complications, improve outcomes, and lower healthcare costs [3]. The pathophysiological rationale was elaborated by Kehlet and Wilmore in 2002 [4]. The ERAS Society was subsequently founded in 2010, building on a first colorectal guideline published in 2005 [5]; the Society has since endorsed guidelines for liver, gastric, esophageal, bariatric, head and neck, and breast reconstruction surgeries [6]. Meta-analytic evidence across specialties consistently demonstrates fewer complications, faster recovery, shorter hospital stays, and improved patient experience with ERAS [7,8].

Introducing ERAS to neurosurgery presents unique challenges due to the brain's complexity and the need for careful neurological monitoring. Formal cranial uptake has trailed colorectal and gastric ERAS by roughly a decade, with the earliest craniotomy-specific ERAS cohorts reported from approximately 2017 onward. Nevertheless, early studies suggest ERAS can be safely and effectively applied in elective craniotomy, typically including preoperative counseling, short fasting, optimized pain and nausea control, reduced use of drains and catheters, early physiotherapy, and a clear discharge and rehabilitation plan. Despite an expanding body of single-study evidence and several prior systematic reviews and meta-analyses on this question [9-10], no ERAS Society guideline yet exists for cranial neurosurgery, and the implementation, workflow, and economic dimensions of cranial ERAS adoption - particularly in low- and middle-income settings - remain comparatively unsynthesized. This review aims to fill that gap alongside summarizing the clinical outcomes evidence.

This narrative review compares ERAS protocols with standard postoperative care in elective craniotomy for brain tumor excision, evaluating outcomes such as hospital length of stay (LOS), complication rates, pain management, and overall recovery, alongside the implementation challenges, workflow barriers, and economic and equity considerations that shape real-world adoption, to support safer, faster, and more patient-centered recovery pathways in neurosurgery.

## Review

Methodology

This is a narrative review, reported according to the Scale for the Assessment of Narrative Review Articles (SANRA) rather than a systematic-review checklist, since no meta-analysis or formal risk-of-bias assessment of individual studies was performed [11]. PubMed/MEDLINE, Embase, Scopus, and Google Scholar were searched from database inception through June 2026 using combinations of the terms "enhanced recovery after surgery," "ERAS," "fast-track," "craniotomy," "brain tumor," "neurosurgery," and "perioperative care." Reference lists of retrieved systematic reviews and meta-analyses were hand-searched for additional eligible studies. Eligible sources included randomized controlled trials, prospective and retrospective cohort studies, and systematic reviews/meta-analyses, in English, addressing perioperative (ERAS or standard) care in adult patients undergoing elective craniotomy; case reports, non-peer-reviewed sources, and studies restricted to non-cranial procedures were excluded unless cited for background context only. Because this is a narrative synthesis, quantitative findings drawn from individual primary studies are reported descriptively, as the range and direction of effects found in the literature, and are not pooled or meta-analytic estimates unless explicitly identified as coming from a cited meta-analysis.

ERAS versus standard postoperative protocols in elective craniotomy

Standardized Protocol for Elective Craniotomy in the Setting of Brain Tumors

**Figure 1:**
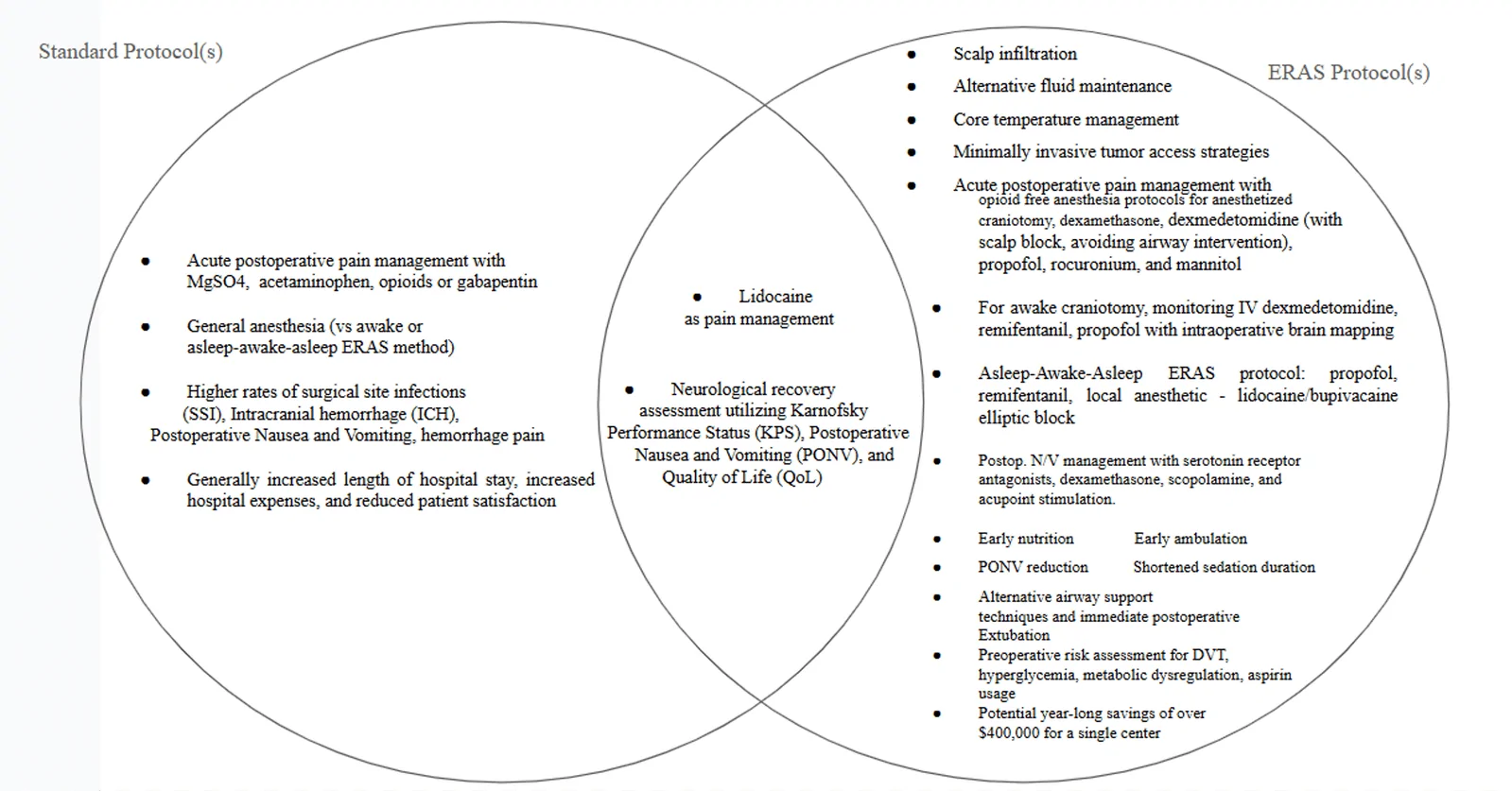
Summary of the differences between the Enhanced Recovery After Surgery (ERAS) protocol versus the standard postoperative care protocol and their intersection. Image credit: Jocelyn N. Wensel. The figure was created using Microsoft PowerPoint (Microsoft Corporation, Redmond, WA).

Understanding the stepwise process for elective craniotomy helps provide context for which the preoperative and postoperative procedures are standardized and practiced by different institutes, as seen in Tables 1-2, for various surgeries. Standardized protocols for elective craniotomy consist of the same general steps (Figure 1).

**Table 1:** Standardized protocols for elective craniotomy in the setting of brain tumors. ERAS, Enhanced Recovery After Surgery; ATPA, anterior transpetrosal approach; HDFT, high-definition fiber tracking

Tumor type	Procedure	Representative institutions/protocols
Gliomas	Awake craniotomy	MD Anderson Cancer Center [21]; Central Hospital of the Irmandade da Santa Casa de Misericordia de São Paolo [22]; Dept. of Psychology, University of Milano-Bicocca and Dept. of Neurosurgery, “S. Chiara” Hospital, Trento [23]; Dept. of Anaesthesia, Jawaharlal Nehru Medical College, Wardha [24]; Siteman Cancer Center [25-26]
	Subtotal vs near-total vs gross-total resections	Mount Lebanon Hospital Univ. Medical Center [27]; Erasmus MC (Rotterdam), Haaglanden MC (The Hague), Univ. Hospital-Leuven, Univ. Hospital-Bern, Massachusetts General Hospital (Harvard), UCSF [28], Chiba University [29], Henry Ford Health [30]
Cavernous and sphenoid wing meningiomas	Orbitozygomatic craniotomy or extended pterional craniotomy with extradural clinoidectomy	Cleveland Clinic Florida [31-33]
Giant intracranial meningiomas	Simpson Grade I-II resection	Univ. of Health Sciences, Gülhane Education and Research Hospital, Ankara [34]
Anterior cranial fossa meningiomas	Subfrontal, pterional, supraorbital, extended endonasal transsphenoidal	Mayo Clinic, Rochester [35]
Petroclival meningiomas	Combined supra- and infratentorial transpetrosal approach (various petrous bone resections with subtemporal-suboccipital craniotomy)	Univ. of Missouri; Barrow Neurological Institute, Phoenix [36]
Intra-axial tumors	Outpatient craniotomy	Toronto Western Hospital [37]
Multiple tumor types	Microscopic anterior transpetrosal approach (ATPA)	Osaka Metropolitan Univ. Graduate School of Medicine [38]; UCLA Health Jonsson Comprehensive Cancer Center and Brain Tumour Center [39]
	Awake craniotomy (variants: hypnosis-assisted, HDFT)	Multiple centers [40-42]

**Table 2:** ERAS protocols for elective craniotomy in the setting of brain tumors. ERAS, Enhanced Recovery After Surgery

Procedure	Representative institutions
ERAS for Elective Craniotomy with Bioelectrical Impedance Analysis	Mayo Clinic Rochester Campus [43]
ERAS for Monitored Anesthetic Care Combined with Scalp Nerve Block in Awake Craniotomy	Barrow Neurological Institute [44]
ERAS for Stereotactic, Awake, Endoscopic, and Extended Bifrontal Craniotomy	Xiangya University Hospital (China) [45]; Pacific Neuroscience Institute, Santa Monica (California); Saint John’s Cancer Institute, Santa Monica (California) [46]; Tangdu Hospital (Xi’an, People’s Republic of China) [47]

**Figure 2:**
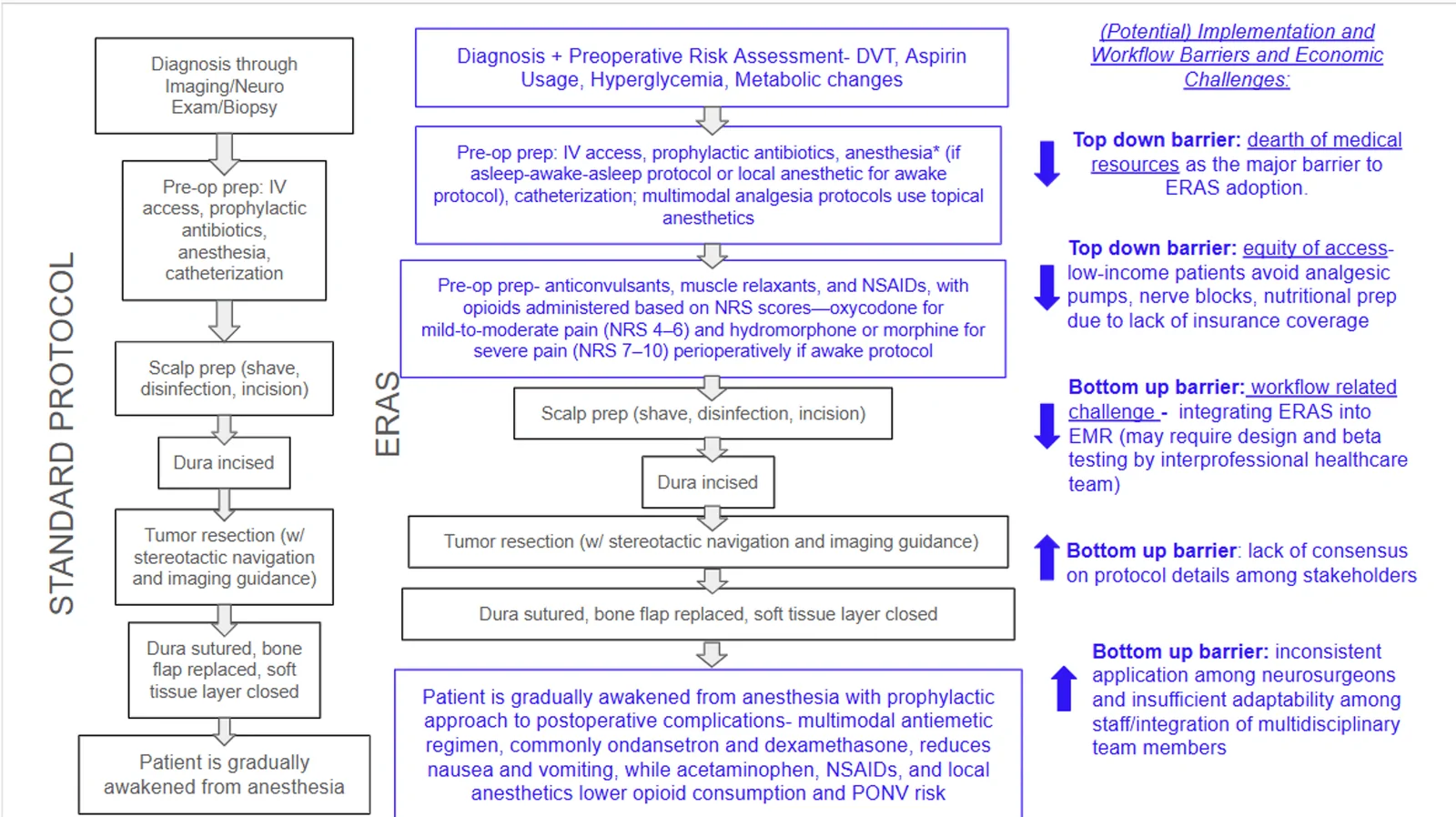
Stepwise processes of the Enhanced Recovery After Surgery (ERAS) protocol and standard postoperative care, with potential implementation and workflow barriers. Image credit: Jocelyn N. Wensel. The figure was created using Microsoft PowerPoint (Microsoft Corporation, Redmond, WA).

Craniotomy for tumor resection usually consists of several standardized steps. After diagnosis through imaging, neurological exam, or biopsy, patients are prepared preoperatively with IV access, antibiotics, anesthesia, and catheterization [1,12-18]. The scalp is shaved, disinfected, and incised to expose the skull, which is opened with a craniotome. Then, the dura is incised to access the brain. Tumor resection is performed, often aided by stereotactic navigation and imaging guidance for precision [2-3,19]. Finally, the dura is sutured, the bone flap is replaced (cranioplasty), soft tissues are closed in layers, and the patient is gradually awakened from anesthesia [6,20]. Institutional and procedural examples of this process, drawn from published series, are summarized in Tables 1-2 [21-47]. The stepwise process is shown in Figure 2.

ERAS Protocol for Elective Craniotomy in the Setting of Brain Tumors

ERAS craniotomy protocols for tumor resection are widely varied and begin with preoperative counseling and comorbidity/risk factor assessment impacting recovery. The Non-Intensive Care (NICE) protocol, developed at Stanford University Medical Center, focuses on preoperative optimization of recovery by modifying craniotomy eligibility screening metrics to assess the likelihood of postoperative cardiopulmonary resuscitation (CPR) in the intensive care unit (ICU), need for intubation, return to the operating room (OR) within 24 hours, mechanical ventilation exceeding four hours, intravenous (IV) vasopressor requirements exceeding 0.4 mg per hour, intracranial hypertension (HTN) requiring cerebrospinal fluid (CSF) drainage and mannitol, dysphagia requiring gastrostomy tube (G-tube) nutrition, or death within 48 hours of surgery. These risks are calculated based on prognostic features such as age, sex, body mass index (BMI), American Society of Anesthesiologists (ASA) classification, Glasgow Coma Scale (GCS) score, neurological screening findings, chronic diseases (diabetes mellitus (DM), HTN, clotting disorders, and seizure disorders), neurosurgical history, anticoagulation use, cardiovascular disease (CVD), and pulmonary disease. Tumor characteristics identified through diagnostic imaging also influence prognosis and eligibility according to the NICE protocol [48].

As noted in the Permanente Journal article, “A 10-Year Analysis of 3693 Craniotomies during a Transition to Multidisciplinary Teams, Protocols, and Pathways,” different centers may adopt varying protocols, including wound care, drain care, neurocritical care, reversal of coagulopathy, DVT prevention, discharge measures, and, in ERAS protocols, early mobilization [49]. Published neurosurgical ERAS recommendations include lesion-specific preoperative counseling, smoking and alcohol cessation for 30 days before surgery (with possible pharmacological or counseling support), and optimized nutrition, such as enteral feeding and carbohydrate-rich clear fluids before the operation. Infection prevention measures include IV cefazolin administration before incision, with vancomycin recommended for patients with a history of methicillin-resistant Staphylococcus aureus (MRSA) [50]. Stress reduction, anxiety and pain management, and prehabilitation (preoperative exercise) are also recommended [51].

Intraoperatively, ERAS emphasizes reduced fluid administration with vasopressors to manage blood flow, contrasting with higher volumes under standard care. Nutrition protocols include preoperative protein and micronutrient support, electrolyte drinks before surgery, and early postoperative feeding beginning with liquids six hours after surgery [45,51].

Postoperative ERAS pain management prioritizes non-opioid analgesia, using agents such as gabapentin, celecoxib, and acetaminophen, along with nonsteroidal anti-inflammatory drugs (NSAIDs) and local nerve blocks. These agents are not associated with high rates of tolerance and dependence as narcotics like morphine or fentanyl [52-53]. Postoperative care also includes neurological assessment, extubation, early ambulation, nausea control, early removal of Foley catheters and IV lines, wound care instruction, and occupational/palliative care support [21,51].

Anesthetic protocol differences and perioperative considerations

Appropriate patient selection, thorough neurological and preanesthetic assessment, and an individualized anesthesia plan enhance recovery and decrease hospital length of stay (LOS) [27,54-55]. Preoperative measures such as counseling, nutrition, antithrombotic medications (antiplatelets/anticoagulants), reversal agents (vitamin K, 4F-PCC, platelet transfusion), and antimicrobial prophylaxis are equally important [27,54-55].

Intraoperative strategies include scalp infiltration, anesthetic protocol, analgesics, fluid maintenance, core temperature management, and minimally invasive tumor access to reduce complications [27,55]. EEG monitoring may be limited near the surgical site, so clinical sedation assessment is more accurate [27,54,56]. Acute postoperative pain is managed with magnesium sulfate, dexmedetomidine, lidocaine, acetaminophen, opioids, or gabapentin [54,55,57]. Dexmedetomidine-based anesthesia with scalp block is increasingly used to avoid airway intervention [21].

Postoperative care includes managing nausea and vomiting with serotonin receptor antagonists, dexamethasone, scopolamine, and acupoint stimulation, which may also provide neuroprotective effects [54,55,58]. Early nutrition and ambulation, along with reduction of postoperative nausea and vomiting (PONV), support faster recovery and fewer complications [55].

Neuroanesthesia principles focus on optimizing operative conditions and maintaining cerebral perfusion pressure (CPP) and oxygenation, with agents such as propofol reducing intracranial pressure (ICP), dexamethasone reducing pain and swelling, and sevoflurane or total intravenous anesthesia (TIVA) with propofol/remifentanil facilitating faster recovery [59-63]. ERAS anesthetic methods differ in sedation depth, agent selection, and airway support [21].

Opioid-free anesthesia protocols using dexmedetomidine, propofol, rocuronium, lidocaine, scalp block, dexamethasone, and mannitol provide stable hemodynamics, sufficient analgesia, reduced nausea and vomiting, and immediate postoperative extubation [62,64]. For awake craniotomy, monitored anesthesia care (MAC) with IV dexmedetomidine, remifentanil, and propofol allows intraoperative arousal for brain mapping and tumor resection [54,56,62,65], while the asleep-awake-asleep (AAA) technique uses propofol, remifentanil, and local anesthesia (elliptic block with lidocaine and bupivacaine) to facilitate seamless transitions between sedation and wakefulness [62,66]. Both approaches improve outcomes and reduce functional complications compared with general anesthesia [54,60].

Concerns about increased bleeding risk have made some neurosurgeons reluctant to use NSAIDs as part of multimodal analgesia after craniotomy. A 2025 systematic review and meta-analysis of 7 studies (2,251 patients) found no significant differences in overall bleeding complications (risk ratio 1.05) or bleeding complications requiring reoperation (risk ratio 1.27) between NSAID and non-NSAID analgesic regimens, supporting selective NSAID use as a reasonable component of opioid-sparing analgesia once hemostasis and coagulation status have been confirmed intraoperatively, rather than as a blanket substitute for opioids in all patients [67]. Regarding seizure prophylaxis, current guidance does not support routine prophylactic anticonvulsants in seizure-naive patients undergoing elective craniotomy for brain tumors; a joint Society for Neuro-Oncology/European Association of Neuro-Oncology update and a Cochrane review both conclude that prophylaxis should be reserved for patients with a seizure history or specific risk factors rather than applied universally, consistent with ERAS's broader preference for minimizing unnecessary pharmacologic burden [68,69].

Preoperative risk assessment and optimization

Extensive preoperative assessment is crucial for clinical outcomes and recovery and is a key part of ERAS protocols. Certain risk factors can significantly affect outcomes in elective craniotomy for brain tumor resection.

Frailty Assessment

Formal frailty scoring is not yet incorporated into a published cranial ERAS protocol in the way the Caprini score is used for VTE, but frailty tools reported in the neuro-oncologic surgery literature include the 5-item modified frailty index (mFI-5) and the Clinical Frailty Scale. In a cohort of brain tumor patients, each one-point increase in mFI-5 was associated with longer ICU and total hospital LOS and higher odds of non-home discharge [70]. Incorporating a validated frailty score into preoperative ERAS risk stratification, alongside the Caprini score, is a reasonable near-term step and represents a current gap in the literature rather than an established component of any published protocol.

Deep Vein Thrombosis

Symptomatic DVT after craniotomy occurs in roughly 1%-10% of patients, while screening-detected (subclinical) DVT on routine duplex ultrasound can approach 30%-50%, depending on the detection method and timing; both are driven by prolonged immobilization, tumor-induced hypercoagulability, and operative time [71-72]. ERAS protocols recommend preoperative VTE risk assessment using tools such as the Caprini score, as shown in Table 3, to inform prophylaxis strategies [73]. Prevention measures include intraoperative and postoperative intermittent pneumatic compression and pharmacological agents, with strict timelines under ERAS protocols compared with standard care. Total Caprini scores map onto four standard risk tiers with corresponding prophylaxis recommendations: very low risk (0-1 point) warrants early ambulation alone; low risk (2 points) warrants mechanical prophylaxis; moderate risk (3-4 points) warrants mechanical prophylaxis with consideration of pharmacologic prophylaxis once hemostasis is confirmed; and high risk (≥5 points) warrants combined mechanical and pharmacologic prophylaxis, reflecting a graded increase in symptomatic VTE incidence across tiers [74].

**Table 3:** Caprini score for risk assessment of venous thromboembolism (VTE) in surgical patients. Updated from [75]. BMI, body mass index; IBD, inflammatory bowel disease; COPD, chronic obstructive pulmonary disease

Risk factor	Points
Age 40-59 years	1
Minor surgery planned	1
BMI ≥ 30 kg/m²	1
History of prior major surgery (<1 month)	1
Swollen legs (current)	1
Varicose veins	1
Sepsis (<1 month)	1
Abnormal pulmonary function (COPD)	1
Acute myocardial infarction (<1 month)	1
Congestive heart failure (<1 month)	1
History of IBD	1
Medical patient at bed rest	1
Pregnant or post-partum	1
History of unexplained or recurrent spontaneous abortion	1
Oral contraceptives or hormone replacement therapy	1
Age 60-74 years	2
Arthroscopic surgery	2
Major open surgery (>45 minutes)	2
Laparoscopic surgery (>45 minutes)	2
Prior cancer (except non-melanoma skin cancer)	2
Present cancer (except breast and thyroid)	2
Confined to bed (>72 hours)	2
Immobilizing plaster cast	2
Central venous access	2
Age ≥ 75 years	3
History of VTE	3
Family history of VTE	3
Present chemotherapy	3
Positive Factor V Leiden	3
Positive Prothrombin 20210A	3
Positive Lupus anticoagulant	3
Elevated anticardiolipin antibodies	3
Elevated serum homocysteine	3
Heparin-induced thrombocytopenia (HIT)	3
Other congenital or acquired thrombophilias	3
Major surgery lasting >6 hours	5
Stroke (<1 month)	5
Elective major lower extremity arthroplasty	5
Hip, pelvis, leg fracture (<1 month)	5
Acute spinal cord fracture or paralysis (<1 month)	5
Multiple traumas (<1 month)	5

Aspirin Usage

Limited studies describe the influence of aspirin and other antiplatelet medications before elective brain surgery. Hanaliouglu et al. found no direct correlation between aspirin usage and hemorrhagic complications, with no significant differences in operative blood loss, hemorrhagic, or thromboembolic events among ASA users, those who stopped aspirin before surgery, and non-users [76]. Other studies similarly concluded that aspirin can be safely continued in patients with high cardiovascular risk before craniotomy, with decisions regarding its use based on tumor type and comorbidities [77-81].

Hyperglycemia

Preoperative risk assessment for hyperglycemia is important due to complications such as blood-brain barrier dysfunction, cerebral edema, tumor growth, and postoperative infections. ERAS protocols recommend screening all patients, including those without diabetes, and implementing strict glycemic control, whereas standard protocols usually screen only patients with diabetes and initiate insulin at glucose levels >180 mg/dL [82,83].

Metabolic Changes

Craniotomy can induce metabolic changes including insulin resistance, lipid oxidation, and protein catabolism. ERAS pre- and postoperative nutritional support, including carbohydrate-rich isotonic solutions preoperatively, reduced preoperative fasting, and early oral feeding, has been shown to reduce catabolism, preserve proteins and glucose levels, and improve recovery outcomes, though effects in craniotomy are less pronounced than in other surgeries [43].

Hyponatremia

Hyponatremia is a distinctive perioperative risk after cranial surgery, arising from either the syndrome of inappropriate antidiuretic hormone secretion (SIADH) or cerebral salt wasting (CSW), and is best characterized in skull-base/pituitary cohorts, where incidence has been reported at approximately 18%, with SIADH accounting for the majority of cases and CSW and desmopressin overuse accounting for the remainder; incidence in general supratentorial tumor craniotomy is less well characterized and likely varies by tumor location and extent of hypothalamic-pituitary axis manipulation [84]. Management follows from etiology: SIADH is managed primarily with fluid restriction, while CSW requires sodium and volume replacement rather than restriction, making serial serum sodium monitoring and accurate etiologic distinction essential in the early postoperative period. ERAS pathways should specify a monitoring schedule (e.g., daily sodium measurements for the first 3-5 postoperative days in higher-risk resections) rather than relying on symptom-triggered testing alone.

Opioid use and patient-reported pain outcomes

Effective pain management is a fundamental component of the ERAS protocol for postoperative care in elective craniotomy for brain tumor excision [85]. Perioperative pain is measured using scales such as the Numerical Rating Scale (NRS) and Visual Analog Scale (VAS), ranging from 0 (“no pain”) to 10 (“worst possible pain”), while other tools include the Behavioral Pain Scale (BPS), Critical Care Pain Observation Tool (CPOT), and Nociception Coma Scale-Revised (NCS-R) [45,58,85]. In standard care, opioids such as fentanyl, sufentanil, remifentanil, and morphine are used continuously, which may cause excessive sedation and delayed detection of ICP changes [86]. In ERAS, multimodal analgesia protocols use topical anesthetics, anticonvulsants, muscle relaxants, and NSAIDs, with opioids administered based on NRS scores: oxycodone for mild-to-moderate pain (NRS 4-6) and hydromorphone or morphine for severe pain (NRS 7-10), as shown in Table 4 [8,45,58,83,85-89]. Scalp blocks and infiltration techniques further reduce opioid requirements [8,58,86]. These approaches improve perioperative outcomes, including lower postoperative complication rates, decreased pain scores, better hemodynamic stability, and reduced opioid-related side effects, while long-term outcomes at discharge and follow-up remain comparable to standard care [8,45,58,83,85-90]. Overall, ERAS pain management enhances early postoperative quality of life (QoL) and reduces hospital LOS compared with traditional protocols [85-87].

**Table 4:** Summary of Enhanced Recovery After Surgery (ERAS) protocol components and associated clinical pain outcomes. NRS, Numerical Rating Scale; VAS, Visual Analog Scale; BPS, Behavioral Pain Scale; CPOT, Critical Care Pain Observation Tool; NCS-R, Nociception Coma Scale-Revised; MAC, monitored anesthesia care; POD, postoperative day; HRQoL, health-related quality of life; QoL, quality of life

Study	ERAS protocol	Tool	Clinical pain outcomes
Wang et al. (2022) [45]	Multimodal analgesia protocol with opioid dose adjustments based on NRS.	NRS, VAS	Lower postoperative pain scores; lower combined opioid-sparing (remifentanil/tramadol) consumption (Standard care: 0.9; ERAS: 0.7) [units and per-agent breakdown to be reconfirmed against the primary source before submission, since a single combined value across two distinct agents is not directly interpretable]).
Kvolik et al. (2022) [58]	Reduced (short-term) opioid usage with non-opioid agents, and regional scalp blocks	BPS, CPOT, NCS-R	Lower postoperative pain scores, improved hemodynamic stability, and reduced opioid-related side effects.
Elayat et al. (2021) [83]	Multimodal analgesia with patient-specific pain control strategies	VAS	Lower postoperative pain scores (POD1 and POD3), lower opioid usage (Fentanyl equal to 100 ug instead of 300 ug).
Qu et al. (2020) [85]	Multimodal analgesia with patient-specific pain control strategies	NRS, VAS	Lower postoperative pain scores (POD1 and POD3), 35-50% reduced opioid use.
Zangi et al. (2024) [86]	Multimodal analgesia with scalp blocks and infiltration techniques	NRS	Lower postoperative pain scores (POD1 and POD3), reduced opioid requirements.
Azghadi et al. (2025) [88]	Multimodal analgesia employed in awake craniotomy under MAC	NRS	Decreased intra-op and post-op opioid usage; improved intraoperative stability.
Qu et al. (2020) [89]	Multimodal analgesia with patient-specific pain control strategies	NRS	Lower postoperative pain scores (POD1 and POD3); lower opioid usage (Fentanyl - Standard care: 226.7 μg; ERAS: 162.5 μg).
Choudhary et al. (2025) [8]	Multimodal analgesia, regional scalp block, opioid minimization	NRS, VAS	Lower postoperative pain scores and reduced opioid-related side effects.
Liu et al. (2020) [90]	ERAS with structured post-op monitoring and pain control	NRS, HRQoL	Comparable long-term pain control in both groups, with better early QoL outcomes within the ERAS group.

Neurological recovery metrics

The need to assess the effectiveness of emerging protocols, such as ERAS, is crucial for their implementation in the field of neurosurgery. Hence, neurological recovery metrics, including the Karnofsky Performance Status (KPS), PONV, and QoL, are utilized in research to yield objective data, used to assess various clinical outcomes that may result from differing protocols. They enable ERAS to be compared with routine care protocols, which are patient management techniques commonly established through the personal experience of the surgical specialists [91]. Hence, standard postoperative care tends to remain non-standardized, leading to inconsistent perioperative care [85]. Therefore, these metrics are pertinent tools to evaluate the efficacy of ERAS, especially for complex neurosurgical procedures, including elective tumor craniotomy. Since one of the aims of ERAS is to accelerate postoperative recovery [91], comparing ERAS with existing protocols in terms of various recovery factors such as competency and overall well-being, nausea and vomiting, and functional capabilities using neurological recovery metrics enables research to validate ERAS and its role in postoperative neurosurgical care.

The KPS is a neurological recovery metric that holistically assesses mortality and independent functional capabilities of postoperative patients [45]. A retrospective study by Feng et al. (*n* = 50) reported a KPS score of 80 in patients managed under standard protocols [91], while ERAS management yielded a 10-point increase maintained at 30 days [45]. A meta-analysis by Choudhary et al. further supported these findings with a statistically significant mean difference of 6.68 points [8]. Not all individual studies show a clinically significant KPS change, underscoring heterogeneity across the primary literature. Another crucial recovery metric is the PONV metric, since nausea is an adverse effect observed in approximately 47% of patients post-craniotomy [54]. The meta-analysis by Choudhary et al. reported an odds ratio of 0.39, signifying a decrease when ERAS was used [8], and Wang et al. reported a reduction from 28.0% to 9.2% between standard-care and ERAS groups, respectively [45]. A clinical trial by Feng et al. also showed that ERAS patients required fewer days of antiemetic medication compared with standard care [91].

To further investigate the role of ERAS in accelerating recovery, the QoL metric [92], specifically HRQoL, is utilized to provide a holistic review of overall health. Liu et al. demonstrated an improvement of 10.8 points in QoL scores in the ERAS group compared with the standard protocol [90]. Levy et al. also reported higher satisfaction in ERAS patients (92.2% vs. 86.8%), with the difference statistically significant [93].

Overall, the neurological recovery metrics, including KPS, PONV, and QoL, enable quantification of outcomes such as independence, nausea-related complications, and well-being. Thus, they allow comparisons between ERAS and standard protocols to evaluate the effectiveness of ERAS in addressing postoperative limitations and inconsistencies in standard care.

Clinical outcomes: ICU versus ward admission and LOS

Elective craniotomy demands a complex and robust team and infrastructure for postoperative recovery, observation, and discharge. According to ERAS protocols, ICU admission should be avoided if possible, and outpatient or same-day discharge surgery has been implemented on a per-center basis. Venkatraghavan et al. reported patients discharged the same day or admitted only to the ward, with none requiring ICU admission [94], and Nassiri et al. similarly found no outpatient conversions, with all inpatients admitted to the ward [95]. Khan et al. used an ERACS protocol and reported higher proportions of same-day elective surgery patients, though not statistically significant [96]. Overall, ERAS protocols reduce ICU LOS to less than a day [97], with studies reporting shorter ICU stays compared to standard care [7,98,99], and Elayat et al. showed reduced need for ICU hospitalization beyond 48 hours (43% vs. 77%) [83]. Newer pathways have also been piloted to bypass ICU admission, such as the safe transition pathway (STP) proposed by Young et al. [98].

Financial impact

Inpatient hospitalization costs are influenced by multiple factors, with ICU requirement being the most impactful. Literature reports per-patient cost reductions between $1,044 and $4,026 [7,89,99]. Individual studies from multiple countries report reductions in overall cost of care ranging from roughly 5% to over 20% [7,51,83,89,92-100]; because these studies differ in currency, costing methodology, and health system context, and no meta-analysis of cost outcomes has been performed, these figures are reported here as the descriptive range identified in individual primary studies rather than as a pooled or meta-analytic estimate. Studies from Thailand, China, the Netherlands, and Spain all demonstrated significant savings with ERAS compared to standard protocols [7,51,85,97,101]. Newer pathways, such as STP, have shown potential year-long savings of over $400,000 for a single center, without differences in complications or readmission rates [98]. While hospitalization costs vary by region, adherence, and available technologies, income levels across economic regions do not appear to alter the financial advantages of ERAS.

Postoperative complications

Complications are inherent in neurosurgery; these can be potentially life-threatening, leading to rapid decline in neurologic function and decreased patient survival. Among the most frequently encountered postoperative complications after elective craniotomies are surgical site infections (SSI), intracranial hemorrhage (ICH), PONV, and hemorrhage pain. These complications cause an increase in Length of hospital stay, increased hospital expenses, and reduced patient satisfaction.

Prophylactic Strategies for Postoperative Complications

ERAS protocols include practices to minimize postoperative complications. A multimodal antiemetic regimen, commonly ondansetron and dexamethasone, reduces nausea and vomiting, while acetaminophen, NSAIDs, and local anesthetics lower opioid consumption and PONV risk [102]. Perioperative antibiotic prophylaxis, typically cefazolin within 60 minutes of incision, with weight-based dosing, re-dosing during prolonged surgeries, and discontinuation within 24 hours, reduces SSIs [103]. In a study by Uribe et al., fewer ERAS patients experienced moderate-to-severe nausea or required antiemetics compared to controls, suggesting improved patient outcomes [104]. PONV rates fall substantially with structured antiemetic prophylaxis and reduced opioid use compared to standard care [105]. Graded compression stockings, intermittent pneumatic compression, and early ambulation decrease DVT risk (Table 5).

**Table 5:** Comparison of postoperative complications between Enhanced Recovery After Surgery (ERAS) and standard protocols.

Postoperative complications	Standard protocol	ERAS protocol
Surgical site infection (SSI)	Standard protocols involve delayed mobilization and limited pre-surgical carbohydrate loading, which increases the risk of SSI. Suboptimal infection control due to a lack of preoperative antibiotic prophylaxis. Incidence of SSI ranges from 2% to 7% [90].	Timely antibiotic administration, early mobilization, and immediate wound debridement result in reduced rates of SSI. In the 2023 meta-analysis, rates remained within 1.5%-3.5% [93].
PONV (postoperative nausea and vomiting)	Use of general anesthesia and opioid based analgesia results in PONV. Lack of strategic antiemetic administration led to increased rates of PONV, as high as 20%-70% [93].	Use of structured antiemetic prophylaxis and reduced opioid use lowers the incidence of PONV. Grant et al. reported a reduction from 50%-70% in standard care to 15%-30% with ERAS protocols [105]. Further improvement ensued with the use of non-opioid analgesics such as NSAIDs, gabapentinoids, and acetaminophen.
Blood loss and transfusion requirement	Improper focus on hemostasis, leading to significant perioperative blood loss requiring transfusions, which are associated with worse outcomes [90].	Controlled hypotension, optimizing hemoglobin levels preoperatively, and restrictive fluid administration result in lower intraoperative blood loss and resulting transfusions [37].
Intracranial hemorrhage (ICH)	Uncontrolled blood pressure during surgery may lead to a severe complication of ICH. Early postoperative anticoagulation results in a worse outcome [90].	Meticulous control of blood pressure during surgery and early neurological assessment to detect signs of ICH. This results in a significant reduction in postoperative ICH [37].
Delayed mobilization and deep vein thrombosis (DVT)	More than a 24-hour delay in mobilization post-surgery leads to DVT and pulmonary embolism [90].	Early mobilization (<24 hours) reduces risks of DVT, pulmonary complications, shortens the length of stay, and improves functional outcomes [93].

Implementation challenges

Implementing ERAS in neurosurgery faces multiple challenges. Time constraints are significant, as much clinical care focuses on acute management, while ERAS requires proactive perioperative interventions like prehabilitation, early patient education, and quick mobilization [104,106]. Although ERAS reduces hospital stays, it increases outpatient demands for follow-up, rehabilitation, and remote monitoring, which may be perceived as burdensome in settings with limited appointment access or rigid protocols [107]. Personnel limitations also pose barriers; effective ERAS implementation requires collaboration among surgical, anesthetic, nursing, dietary, and rehabilitation teams, which is more feasible in large academic centers but challenging in smaller or underfunded facilities that lack formal neuro-rehabilitation infrastructure [108-109]. Without adequate support, additional ERAS responsibilities may contribute to physician fatigue and burnout [110]. Knowledge gaps further impede adoption, as many physicians outside gastrointestinal or gynecological care remain unfamiliar with ERAS applications, and neurosurgeons may be uncertain about its mechanisms in procedures such as spinal fusion, intracranial surgery, or post-stroke recovery [105]. Patients also often lack adequate information and preparation, which are critical for early discharge and mobility; insufficient education can lead to protocol noncompliance and adverse outcomes [111]. Comprehensive ERAS education should include bedside instruction, accessible patient resources, and systematically arranged outpatient support.

Economic and workflow barriers to ERAS adoption

In addition to the implementation challenges discussed above, the adoption of ERAS protocols can be difficult due to considerable economic and workflow-related obstacles.

Economic Barriers

The scarcity of resources can be a significant barrier to ERAS adoption. As a result, ERAS protocols are less likely to be implemented in public practice settings with limited resources, as reported by Di Donato et al. [99]. In countries such as Malaysia, resource limitations result in minimal ERAS adoption in both private and public institutions [99]. In a multicenter study in China, Wang et al. reported that all 42 participants (surgeons, anesthesiologists, nurses, and dietitians) identified the dearth of medical resources as the major barrier to ERAS adoption [112]. In low- and middle-income countries, this scarcity can be particularly severe; for example, in Brazil, where 65 percent of the population depends on the public healthcare system, only 45 percent of public spending is devoted to it, leading to resource shortages [99].

Cost issues can also affect individual patients. In Kenya, ERAS protocols may increase treatment costs because patients must cover hospital care expenses [99]. Similar concerns exist in China, where some low-income patients avoid analgesic pumps, nerve blocks, or nutritional preparations due to lack of insurance coverage, negatively impacting postoperative care [112].

However, not all studies report increased costs. Supbumrung et al. found that ERAS implementation reduces hospitalization costs by an average of $1,188 per patient [113]. While acknowledging resource limitations in developing countries, they emphasize that ERAS reduces both LOS and healthcare costs in developing and developed settings. Similarly, Riad et al. suggest that ERAS adoption in low- and middle-income countries is feasible and decreases LOS [114].

Workflow Barriers and Persistence of Standard Postoperative Care

The success of ERAS largely depends on effective and constant communication among multidisciplinary team (MDT) members throughout the perioperative period. A major difficulty in implementation is the lack of collaboration and efficient communication between MDTs, as suggested by Wang et al. and Lyon et al. [112,115]. Agarwal et al. identified four workflow-related challenges: integrating ERAS into electronic medical record (EMR) systems, lack of consensus on protocol details among stakeholders, inconsistent application among neurosurgeons, and insufficient adaptability among staff [116].

Given these workflow and economic challenges, standard postoperative care remains common, especially in low- and middle-income countries where resources are limited [94]. ERAS also increases workloads for doctors and nurses, which may not be compensated with financial incentives, particularly in less wealthy countries [112]. Patients may resist ERAS due to higher treatment costs, and the inherently multidisciplinary nature of ERAS requiring coordination among anesthesiologists, nurses, and surgeons further complicates implementation, indirectly encouraging the continuation of standard care [112,116].

Research scarcity and technologies

Kehlet and Wilmore defined the essential mechanism of postoperative complications as the degree of pathophysiological stress response to surgery and subsequent organ dysfunction [4,109]. That response is a major factor impacting outcome and recovery, making the need for ERAS protocols pivotal. Despite being patient-tailored, ERAS faces challenges [107], including research gaps from setting heterogeneity, protocol variations, and lack of unified standards that impede compliance and invalidate comparisons [117-118]. ERAS protocols have more traction in high-income countries [119], while studies in Pakistan identified barriers such as limited interdisciplinary collaboration, surgeon knowledge, and poor communication across patients, doctors, and staff [119-120]. Patient compliance is another challenge [112], especially for measures like smoking cessation and alcohol abstinence. To address this, AI and wearable devices have been proposed to improve compliance, with telemedicine interventions reinforcing adherence [117,121]; applications such as MySurgeryRisk can also predict postoperative outcomes with high sensitivity and specificity [122]. Although ERAS protocols vary between specialties, their benefits are clear, and effective implementation begins with the surgeon’s first encounter with the patient [123].

## Conclusions

The implementation of enhanced recovery protocols for craniotomy demonstrates tangible benefits when compared to standard protocols. Patients benefit from reductions in complications and LOS, reduced disability, and improved recovery. Neurosurgeons have access to newer surgical approaches and techniques, with the possibility of safe same-day discharge. Multidisciplinary approaches streamline the hospital stay from admission to discharge while improving symptom control and reducing the physical, emotional, and psychological strain from surgery. The economic burden for hospitals can be reduced as ERAS provides cost-effective alternatives without sacrificing patient safety and satisfaction. Although evidence illustrates the benefits of enhanced recovery, its global implementation is hindered by regional practices, institutional policies and protocols, resource scarcity, and inequality. Making ERAS protocols the new standard of care can improve global outcomes; further research is warranted, especially in middle- to low-income settings where the limitations of these protocols are most prominent.
